# The use of interdental cleaning devices and periodontal disease contingent on the number of remaining teeth in Korean adults

**DOI:** 10.1038/s41598-022-17885-7

**Published:** 2022-08-16

**Authors:** Yun-Jeong Kim, Yoon Min Gil, Kwang-Hak Bae, Seon-Jip Kim, Jungjoon Ihm, Hyun-Jae Cho

**Affiliations:** 1 Department of Periodontology, Seoul National University Gwanak Dental Hospital, Seoul, Republic of Korea; 2 Dental Research Institute, School of Dentistry, Seoul National University, Seoul, Republic of Korea; 3 Department of Dentistry, School of Dentistry, Seoul National University, Seoul, Republic of Korea; 4 Seoul SUN Dental Hospital, Paju-si, Gyeonggi-do Republic of Korea; 5 Department of Dental Education, School of Dentistry, Seoul National University, 1 Gwanak-Ro, Gwanak-Gu, Seoul, 08826 Republic of Korea; 6 Department of Preventive Dentistry and Public Oral Health, School of Dentistry, Seoul National University, 101 Daehak-Ro, Jongno-Gu, Seoul, 03080 Republic of Korea

**Keywords:** Epidemiology, Dentistry, Public health

## Abstract

This study aimed to investigate the effect of interdental brushes and dental floss on the prevention of periodontitis in participants with ≥ 20 or < 20 remaining teeth by using the Korea National Health and Nutrition Examination Survey 2016–2018. Data from 11,614 participants were analysed using multivariate logistic regression after adjusting for sociodemographic factors (age and sex), socioeconomic factors (level of education and individual income), oral health-related variables (daily toothbrushing), and systemic health-related variables (smoking, diabetes, and obesity). The adjusted odds ratio (AOR) showed statistically significant results for both floss (AOR, 1.41; 95% confidence interval (CI) 1.22–1.64) and interdental brushes (AOR, 1.16; 95% CI 1.01–1.34). However, no significant difference was found in the subjects with fewer than 20 teeth. The subgroup analysis showed that interdental brushes had a significant preventive effect on women who had more than 20 teeth. Among participants with fewer than 20 teeth, interdental brush users had more periodontitis in men. Regarding those with more than 20 teeth, health inequality was alleviated when floss and interdental brushes were used. The bottom line is that the effect of preventing periodontitis in interdental brushes and dental floss was more evident in participants with ≥ 20 remaining teeth rather than in participants with < 20 remaining teeth.

## Introduction

Bacterial plaque in the form of biofilms that remain primarily in the interdental area even after tooth brushing can cause periodontal tissue inflammation. To prevent this condition, different cleaning methods, such as dental flossing and interdental brushing, are applied to effectively remove plaques^1^. The American Dental Association recommends self-cleaning of the interdental area to control periodontal disease, a problem common in the population^2^. Numerous studies on interdental cleaning have shown that dental floss, interdental brushes, and water flossers can effectively remove plaque that remains between teeth^3–7^.

However, data about the effects of dental flossing and interdental brushing on oral health and periodontal disease prevention are insufficient^8,9^. Based on a recent large-scale longitudinal study, interdental cleaning could alleviate self-reported gingivitis. However, its effectiveness in preventing advanced periodontitis has not been confirmed^10^. According to the study of Kim and Han, dental floss, but not interdental brushes, could prevent periodontal disease^8^. Thus, a stronger and more reliable approach is needed, as the efficacy of these interdental hygiene tools in the overall population is not clearly elucidated.

The prevalence of severe periodontitis among elderly individuals can be masked by a high frequency of edentulism and a large number of missing teeth^11,12^. In fact, several studies have assessed the importance of more than 20 teeth when used as an explanatory or outcome variable^13–15^. Based on the Korea National Health and Nutrition Examination Survey (KNHANES), there was a significant difference in the prevalence of periodontitis according to the presence of more than 20 teeth among floss and interdental brush users (Supplementary Fig. S1). Therefore, it is necessary to examine the prevention effect of periodontitis in interdental cleaners by dividing them into subjects with 20 or more teeth and subjects with less than 20 teeth. However, most studies assessed the efficacy of interdental cleaners in the overall population without considering the number of remaining teeth.

Hence, this study aimed to investigate the effect of interdental brushes and dental floss on periodontitis prevention among healthy adults with 20 or more remaining teeth or 19 or fewer remaining teeth using KNHANES.

## Results

In total, 11,614 adults aged 19 years or older were eligible for this study. All variables had a number of different missing values, so the total number by each variable is different. Table 1 shows their characteristics. In the group with 20 or more teeth, 83.4% and 85.6% of participants with periodontitis did not use dental floss and an interdental toothbrush, respectively. In the group with fewer than 20 teeth, 93.4% and 87.6% of participants with periodontitis did not use dental floss and an interdental toothbrush, respectively. Additionally, 11.4% of participants with periodontitis in the group with 20 or more teeth and 23.1% of participants with periodontitis in the group with less than 20 teeth brushed their teeth less than once a day.

**Table 1:** Characteristics of the study population stratified by periodontitis.

Subjects with variable^b^	20 or more teeth				*p* value	Less than 20 teeth				*p* value
	Normal		Periodontitis^a^			Normal		Periodontitis^a^		
	n	Weighted%	n	Weighted%		n	Weighted%	n	Weighted%	
Number of natural teeth^c^	7815	26.77 ± 0.03	3199	25.79 ± 0.05	< 0.001	893	11.49 ± 0.21	782	13.10 ± 0.21	< 0.001
Age^c^	7815	44.25 ± 0.28	3199	56.53 ± 0.33	< 0.001	893	69.54 ± 0.39	782	68.24 ± 0.46	0.017
**Sex**										
Male	3051	37.6	1710	51.6	< 0.001	366	39.9	418	54.1	< 0.001
Female	4764	62.4	1489	48.4		527	60.1	364	45.9	
**Income**										
Low	2044	22.8	842	26.5	< 0.001	262	28.9	252	32.7	0.418
Medium–low	2183	24.1	832	25.7		241	25.5	213	26.5	
Medium–high	2291	26.0	781	24.0		216	25.0	180	23.0	
High	2366	27.1	742	23.7		175	20.5	135	17.8	
**Education**										
≤ Elementary school	1278	13.7	757	24.8	< 0.001	478	54.7	392	50.6	0.289
Middle school	971	11.0	404	14.1		116	13.9	127	17.8	
High school	2723	32.8	999	32.5		172	22.1	135	21.2	
≥ University or college	3612	42.5	897	28.6		74	9.2	71	10.4	
**Flossing**^**d**^										
No	6330	71.1	2659	83.4	< 0.001	812	92.4	719	93.4	0.514
Yes	2512	28.9	521	16.6		67	7.6	48	6.6	
**Interdental brushing**^**d**^										
No	6997	78.8	2695	85.6	< 0.001	785	89.2	671	87.6	0.431
Yes	1845	21.2	485	14.4		94	10.8	96	12.4	
**Daily toothbrushing**										
Once or none	652	6.8	372	11.4	< 0.001	180	19.0	179	23.1	0.074
Twice or more	8190	93.2	2808	88.6		698	81.0	587	76.9	
**Smoking**										
Current	1187	13.3	789	23.8	< 0.001	116	13.8	180	23.9	< 0.001
Past	1412	15.9	777	24.0		222	23.2	211	27.2	
Never	6245	70.9	1613	52.2		541	63.0	376	48.9	
**Diabetes**^**e**^										
Normal	511	6.6	547	17.6	< 0.001	190	22.7	218	29.7	0.023
Impaired fasting glucose	1482	20.1	921	29.0		243	31.7	198	27.2	
Diabetes	5434	73.3	1559	53.4		359	45.5	300	43.1	
**Body mass index**^f^										
Underweight	2607	28.9	1351	40.5	< 0.001	327	36.6	280	36.1	0.966
Normal	5658	64.5	1783	57.5		536	60.1	468	60.7	
Obese	619	6.7	71	2.0		27	3.4	27	3.2	

^a^Periodontitis was defined as community periodontal index codes 3 and 4.

^b^All variables have a number of different missing values, so the total number by each variable is different.

^c^Continuous variables are denoted by the mean ± standard error.

^d^Daily use of interdental brush and dental floss.

^e^Impaired fasting glucose was defined as 100 mg/dL ≤ fasting blood glucose < 126 mg/dL, and diabetes was defined as fasting blood glucose ≥ 126 mg/dL or current use of antidiabetic drugs or insulin.

^f^Obesity status was defined as underweight (< 18.5 kg/m^2^), normal (18.5–24.9 kg/m^2^), and obese (≥ 25.0 kg/m^2^).

Table 2 shows the results of the logistic regression analysis of the association between the use of interdental cleaners and periodontitis. In the group with 20 or more teeth, in models 1–4, participants who did not use dental floss had a higher adjusted odds ratio (AOR) for periodontitis (model 1 AOR: 1.55, 95% CI 1.35–1.78; model 2 AOR: 1.44, 95% CI 1.26–1.66; model 3 AOR: 1.44, 95% CI 1.25–1.66; and model 4 AOR: 1.41, 95% CI 1.22–1.64). Regarding interdental brushing, in model 4, participants who did not use an interdental brush had a significantly higher risk of periodontitis than those who used an interdental brush (model 4 AOR: 1.16, 95% CI 1.01–1.34). However, in the group with fewer than 20 teeth, there was no significant difference between groups in all models 1–4 in flossing or interdental brushing.

**Table 2:** Multivariable association between interdental cleaner use and periodontitis.

AOR (95% CI)	Model 1		Model 2		Model 3		Model 4	
Number of teeth	≧ 20	< 20	≧ 20	< 20	≧ 20	< 20	≧ 20	< 20
Number of subjects	10,930	1641	10,514	1549	10,514	1548	10,009	1405
**Flossing**								
No	**1.55 (1.35–1.78)**	1.23 (0.78–1.93)	**1.44 (1.26–1.66)**	1.17 (0.74–1.85)	**1.44 (1.25–1.66)**	1.17 (0.74–1.85)	**1.41 (1.22–1.64)**	1.19 (0.74–1.90)
Yes	Reference		Reference		Reference		Reference	
**Interdental brushing**								
No	1.13 (0.99–1.30)	0.85 (0.58–1.23)	1.11 (0.96–1.28)	0.85 (0.57–1.28)	1.11 (0.96–1.28)	0.85 (0.57–1.27)	**1.16 (1.01–1.34)**	0.82 (0.53–1.26)
Yes	Reference		Reference		Reference		Reference	

*AOR* adjusted odds ratio, *CI* confidence interval.

Bold denotes statistical significance at P < 0.05.

Response variable: Periodontitis.

Explanatory variable: Interdental cleaner.

Model 1 was adjusted for age and sex.

Model 2 was adjusted for age, sex, household income, and educational level.

Model 3 was adjusted for age, sex, household income, educational level, and daily toothbrushing.

Model 4 was adjusted for age, sex, household income, educational level, daily toothbrushing, smoking, diabetes mellitus, and BMI.

Table 3 shows the results of the subgroup analysis of the association between the use of interdental cleaners and periodontitis according to sex and age. In the group with 20 or more teeth, males, females, and participants in their twenties, forties, fifties, and seventies who did not use dental floss had significantly higher risks of periodontitis. Among participants who did not use interdental brushes, females and participants in their thirties and sixties had significantly higher risks of periodontitis. In the group with fewer than 20 teeth, only the male group who did not use interdental brushes had significantly lower risk of periodontitis.

**Table 3:** Subgroup analysis of the association between interdental cleaners and periodontitis by sex and age.

Number of teeth	Number of subjects		Floss (AOR (95% CI), Reference = Yes)		Interdental brush (AOR (95% CI), Reference = Yes)	
	≧ 20	< 20	≧ 20	< 20	≧ 20	< 20
**Sex**						
Male	4334	658	**1.30 (1.05–1.62)**	1.34 (0.67–2.68)	1.04 (0.84–1.28)	**0.28 (0.13–0.61)**
Female	5675	747	**1.51 (1.25–1.82)**	1.28 (0.67–2.46)	**1.27 (1.03–1.56)**	1.58 (0.95–2.65)
**Age**						
19–29 years old	1439	1	**3.42 (1.31–8.95)**	**–**	0.62 (0.34–1.12)	**–**
30–39 years old	1898	8	1.11 (0.77–1.60)	–	**1.45 (1.01–2.09)**	–
40–49 years old	2185	39	**1.66 (1.26–2.20)**	**–**	1.11 (0.84–1.48)	**–**
50–59 years old	2070	184	**1.47 (1.13–1.92)**	1.36 (0.42–4.37)	1.06 (0.80–1.42)	0.38 (0.11–1.36)
60–69 years old	1509	440	1.26 (0.88–1.80)	1.48 (0.64–3.45)	**1.69 (1.14–2.51)**	1.18 (0.56–2.52)
Over than 70 years old	908	733	**1.84 (1.06–3.21)**	0.82 (0.37–1.84)	0.89 (0.53–1.49)	0.66 (0.36–1.19)

*AOR* adjusted odds ratio, *CI* confidence interval.

Bold denotes statistical significance at P < 0.05.

–: Logistic regression analysis is not possible due to insufficient number of subjects.

Table 4 shows the results of the stratified analysis of the association between individual income and educational level and periodontitis according to the use of dental floss and interdental brushes. Participants with low and medium–low incomes who did not use both dental floss and an interdental brush were at significantly higher risk of periodontitis than those with high individual incomes. Furthermore, participants who attained elementary school-level qualification or lower and middle school-level qualification had a significantly higher risk of periodontitis than those with a university or college degree or higher. However, there was no significant difference in the risk of developing periodontitis according to individual income and educational level among participants who used only an interdental brush or both dental floss and an interdental brush.

**Table 4:** Multivariable association between socioeconomic status and periodontitis in the entire sample stratified by floss and interdental brush.

Characteristic	Total	No use of floss or an interdental brush	Only floss use	Only interdental brush use	Both
	AOR (95% CI)	AOR (95% CI)	AOR (95% CI)	AOR (95% CI)	AOR (95% CI)
Number of Subjects	10,009	6070	1904	1221	814
**Income**					
Low	**1.34 (1.12**–**1.60)**	**1.33 (1.09**–**1.62)**	1.44 (0.93–2.22)	1.42 (0.88–2.29)	1.11 (0.62–1.99)
Middle low	1.18 (1.01–1.39)	**1.22 (1.01**–**1.48)**	1.18 (0.78–1.79)	1.23 (0.75–2.04)	0.57 (0.29–1.12)
Middle high	1.11 (0.96–1.29)	1.11 (0.93–1.32)	1.32 (0.88–1.97)	1.08 (0.70–1.65)	0.71 (0.41–1.25)
High	Reference	Reference	Reference	Reference	Reference
**Education**					
≤ Elementary school	**1.34 (1.09**–**1.65)**	**1.37 (1.08**–**1.75)**	1.52 (0.76–3.05)	0.79 (0.40–1.55)	1.72 (0.46–6.43)
Middle school	**1.26 (1.01**–**1.59)**	**1.31 (1.01**–**1.69)**	1.16 (0.61–2.20)	1.01 (0.51–1.97)	0.86 (0.25–3.05)
High school	**1.20 (1.04**–**1.39)**	1.15 (0.97–1.37)	**1.48 (1.06**–**2.07)**	0.96 (0.66–1.41)	1.43 (0.85–2.39)
≥ University or college	Reference	Reference	Reference	Reference	Reference

*AOR* adjusted odds ratio, *CI* confidence interval.

Bold denotes statistical significance at P < 0.05.

## Discussion

Periodontitis is a chronic inflammatory disease in which supporting tissues around the teeth are destroyed by infection and the host immune response due to bacterial plaque accumulation^16^. Periodontal breakdown could be a major cause of tooth loosening with ageing and could affect oral functions such as mastication and pronunciation^11,13,16–18^. Approximately half of American adults aged over 30 years have periodontitis^18,19^. In Korea, according to counts of the frequency of outpatient treatment by the Health Insurance Review and Assessment Service (HIRA), gingivitis and periodontal disease have shown the highest rates of diseases since 2019^20^.

Toothbrushing is a fundamental self-care behavioural activity for maintaining oral health. Frequent brushing is effective at reducing the formation of biofilms^21^. However, a meta-study found that the actual plaque removal rate was only 42%^22^. Thus, thoroughly cleaning the interdental area where plaque mainly accumulates using various tools, such as dental floss and interdental brushes, is commonly recommended as a standard procedure for oral hygiene management. Education on interdental cleansers by dentists and dental hygienists is important, but in reality it is not properly applied in the treatment field^23^.

A recently published nationally representative longitudinal study revealed that frequent interdental cleaning is associated with better self-perceived oral health and less volume of gingival bleeding but less with measures of more advanced periodontal disease^10^. The proportion of participants who used interdental cleaners in NHANES from United States exceeded 70%. However, the fundamental background may be different from the Korean data, which show a usage rate of 10%–20% for interdental cleaners. One possibility is that more people in Korea may use interdental cleaners to remove food stuck between teeth, not dental biofilm, for prevention. Hence, we divided participants into those with ≥ 20 and < 20 remaining teeth. Our logistic regression analysis revealed that the risk of periodontitis was evidently high among participants with ≥ 20 remaining teeth who did not use floss and interdental brushes.

Several studies have reported that daily flossing can reduce the prevalence of gingivitis and periodontitis^4,24^, thereby emphasizing that interdental cleaning is extremely effective^7,25^. In particular, interdental brushing is considered the most effective method for cleaning between teeth^26^ and reduces periodontal pathogens between teeth^27^. In a 2015 meta-study, Sälzer found moderate evidence about the efficacy of using an interdental brush combined with brushing compared with toothbrushing alone. That is, there was a 34% reduction in gingivitis and a 32% decrease in plaque scores^7^.

This study showed that interdental brushing and flossing were effective among young and healthy subjects. Based on the subgroup analysis according to sex and age, the female, twenties, and sixties groups were more likely to have periodontitis if they did not use interdental brushes. Interestingly, in the male group with fewer than 20 teeth, interdental users had a higher risk of periodontal disease. This is consistent with our reasoning that interdental brushes are used for food removal rather than preventive behaviour in those who have < 20 remaining teeth.

Hence, the use of proper interdental hygiene tools is more important than simple use. Generally, interdental brushing is useful only for elderly individuals with enlarged spaces between teeth. However, based on the study of Carrouel^25^, an appropriate-diameter tool must be used according to the size of the interdental space, even among young, healthy individuals. This result is in accordance with that of our cross-sectional study.

In participants who used interdental brushes alone or both dental floss and interdental brushes, there was no significant difference in the risk of periodontitis according to socioeconomic status, particularly personal income and educational background, as shown in Table 4. These results could be an extension of a previous study^2^, which shows that the use of an interdental brush alleviates periodontal health inequalities, and more detailed conclusions could be obtained by performing an analysis using dental floss as a major variable.

The current study had several limitations. That is, specific information about the use of tools, such as the frequency and duration of applying dental floss or interdental brush, was not confirmed. Additionally, as this study was cross-sectional in nature, the effect of interdental cleaning on preventing periodontal disease was not directly analysed, and the assessment of inflammation was only based on the depth of the periodontal pocket (> 3.5 mm) according to the CPI index. As the study design of Pitchika et al. was a repeated cross-sectional study, it is necessary to conduct a study with a similar design when our KNHANES data can be viewed through follow-up observation in the future^28^.

However, the study, which performed a large-scale sample analysis representing the whole population of Korea, also had some strengths. In this study, participants were divided into those with more than 20 remaining teeth and those with less than 20 remaining teeth, thereby confirming the substantial relationship between interdental cleaning and periodontal disease. Significant results were obtained by examining the relationship between periodontitis and socioeconomic status, particularly income and educational background, according to the use of dental floss and interdental brushes.

In conclusion, we found that both interdental brushing and flossing have a cross-sectionally significant periodontitis prevention and inequality alleviation effect in the population with 20 or more remaining teeth.

## Methods

### Data source

This study used nationwide cross-sectional data from the seventh KNHANES conducted between 2016 and 2018. KNHANES has been implemented by the Korea Centers for Disease Control and Prevention since 1998 to assess the health and nutritional status of Koreans. The target population of KNHANES is noninstitutionalized Korean citizens residing in Korea. Among them, approximately 10,000 individuals aged 19 years and over are selected annually using multistage clustered probability sampling. KNHANES collects different health and nutrition data using three types of surveys: health interviews, health examinations, and nutrition assessments. The 2016 and 2017 surveys were conducted without deliberation as they corresponded to a research conducted by the government for public welfare. The 2018 survey was performed with the approval of the institutional review board of the Korea Centers for Disease Control and Prevention with consideration of the collection of human-derived materials and the provision of raw data to a third party (IRB no.: 2018-01-03-P-A), and it was conducted according to the principles of the Declaration of Helsinki; written informed consent was obtained from all subjects^29^. In total, 24,269 individuals participated in the seventh KNHANES. This cross-sectional study conformed to the Strengthening the Reporting of Observational Studies in Epidemiology (STROBE) statement guidelines for the analysis of observational data. In this study, the data of 11,614 participants who were aged 19 years or older were analysed.

1. Assessment of periodontitis (outcome variable).
  The periodontal status of the participants was assessed using the community periodontal index (CPI): 0 = healthy, 1 = bleeding on probing, 2 = calculus formation, 3 = pocket measuring 4–5 mm, and 4 = pocket measuring 6 mm or more^30^. The index teeth used in periodontal examination were the two molars in each posterior sextant, the upper right central incisor, and the lower left central incisor. A trained dentist performed periodontal examination using the World Health Organization CPI periodontal probe, and the highest score was recorded for the sextant. A CPI score of 0, 1, or 2 indicated the absence of periodontitis, and a CPI score of 3 or 4 suggested the presence of periodontitis. CPI was conducted by a trained dentist, and the kappa index ranged from 0.848 to 1.000.
2. Assessment of interdental cleaners (explanatory variable).
  A questionnaire was used to assess whether the participants used dental floss and interdental brush. For the questionnaire item “Please select all products that you use for your oral health other than toothpaste and a toothbrush,” the participants answered yes or no.
3. Covariates.
  The covariates in this study included sociodemographic factors, socioeconomic factors, oral health-related variables, and systemic health-related variables. The sociodemographic factors were age and sex, and the socioeconomic factors were individual income and educational level. Individual income was divided into four quartile groups: low (< 25%), medium–low (25%–49%), medium–high (50–74%), and high (75–100%). Educational level was classified as below elementary school, middle school, high school, and university or college.

The oral health-related variables included daily toothbrushing. Daily toothbrushing was categorized into two groups based on frequency: once or none and twice or more.

The systemic health-related variables included smoking, the presence of diabetes mellitus, and obesity. Smoking status was categorized into three groups based on smoking experience: current, past, and never. Diabetes mellitus was divided into three groups: normal, impaired fasting glucose (100 mg/dL ≤ fasting blood glucose < 126 mg/dL), and diabetes (fasting blood glucose of ≥ 126 mg/dL or the current use of antidiabetic drugs or insulin). Obesity was categorized into three groups using body mass index (BMI) based on the World Health Organization’s redefined obesity criteria for Asia–Pacific regions: underweight (< 18.5 kg/m^2^), normal (18.5–24.9 kg/m^2^), and obese (≥ 25.0 kg/m^2^).

### Statistical analysis

Considering the complex survey design of KHNANES VII, the weighted data were analysed statistically. Age was a continuous variable, and all variables, except age, were categorical variables. The use of interdental cleaner was considered an explanatory variable, and periodontitis was considered a response variable. The characteristics of the study population were analysed according to frequency, weight proportions, and confidence interval (CI) with respect to sociodemographic factors, personal health practices, and general health status. The difference between groups with and without periodontitis according to each variable was determined using a chi-squared test.

The association between the use of interdental cleaner and periodontitis was analysed via multivariate logistic regression analysis. Regression model 1 was adjusted for age and sex. Socioeconomic variables such as individual income and educational level were added to regression model 2. Oral health-related variables, such as daily toothbrushing, were added to regression model 3. Systemic health-related variables such as smoking, diabetes mellitus, and obesity were added to regression model 4. Stratified analyses based on sex, age, individual income, and educational level were conducted to examine the association between the use of an interdental cleaner and periodontitis according to subgroups. All statistical complex sample analyses were conducted using Statistical Package for the Social Sciences version 23.0 (IBM, NY, USA). A *P* value of < 0.05 was considered statistically significant.

## Supplementary Information

Supplementary Figure S1.

## Data Availability

The dataset generated and analysed during the current study and corresponding syntax are available from the first author upon reasonable request.
