# Author Correction: The use of interdental cleaning devices and periodontal disease contingent on the number of remaining teeth in Korean adults

**DOI:** 10.1038/s41598-022-19432-w

**Published:** 2022-09-07

**Authors:** Yun-Jeong Kim, Yoon Min Gil, Kwang-Hak Bae, Seon-Jip Kim, Jungjoon Ihm, Hyun-Jae Cho

**Affiliations:** 1 Department of Periodontology, Seoul National University Gwanak Dental Hospital, Seoul, Republic of Korea; 2 Dental Research Institute, School of Dentistry, Seoul National University, Seoul, Republic of Korea; 3 Department of Dentistry, School of Dentistry, Seoul National University, Seoul, Republic of Korea; 4 Seoul SUN Dental Hospital, Paju-si, Gyeonggi-do Republic of Korea; 5 Department of Dental Education, School of Dentistry, Seoul National University, 1 Gwanak-Ro, Gwanak-Gu, Seoul, 08826 Republic of Korea; 6 Department of Preventive Dentistry and Public Oral Health, School of Dentistry, Seoul National University, 101 Daehak-Ro, Jongno-Gu, Seoul, 03080 Republic of Korea

Correction to: *Scientific Reports* 10.1038/s41598-022-17885-7, published online 16 August 2022

The original version of this Article omitted affiliation for Jungjoon Ihm. The correct affiliations are listed below.

Dental Research Institute, School of Dentistry, Seoul National University, Seoul, Republic of Korea

Department of Dental Education, School of Dentistry, Seoul National University, 1 Gwanak-Ro, Gwanak-Gu, Seoul, 08826, Republic of Korea

The original Article has been corrected.

