# Supplementary material for: The use of interdental cleaning devices and periodontal disease contingent on the number of remaining teeth in Korean adults
Source: Sci Rep. 2022 Aug 16;12:13853. doi: 10.1038/s41598-022-17885-7 (PMC9381548; doi:10.1038/s41598-022-17885-7)
Supplement: Supplementary file 1 — Supplementary Figure S1. [file 41598_2022_17885_MOESM1_ESM.docx]

**The use of interdental cleaning devices and periodontal disease contingent on the number of remaining teeth in Korean adults**

Yun-Jeong Kim, Yoon Min Gil, Kwang-Hak Bae, Seon-Jip Kim, Jungjoon Ihm, Hyun-Jae Cho

Supplementary Figure S1.

Prevalence (%) of periodontitis according to the use of interdental cleaner with with ≥20 remaining teeth versus <20
